# Supplementary material for: Association between oral microbiome and seven types of cancers in East Asian population: a two-sample Mendelian randomization analysis
Source: Front Mol Biosci. 2023 Nov 21;10:1327893. doi: 10.3389/fmolb.2023.1327893 (PMC10702768; doi:10.3389/fmolb.2023.1327893)
Supplement: Supplementary file 1 [file DataSheet1.ZIP › Supplementary Materials/MR plots of saliva/saliva═╝/colorectal cancer/pheno.2134_to_colorectal cancer_leave_one_out.pdf]

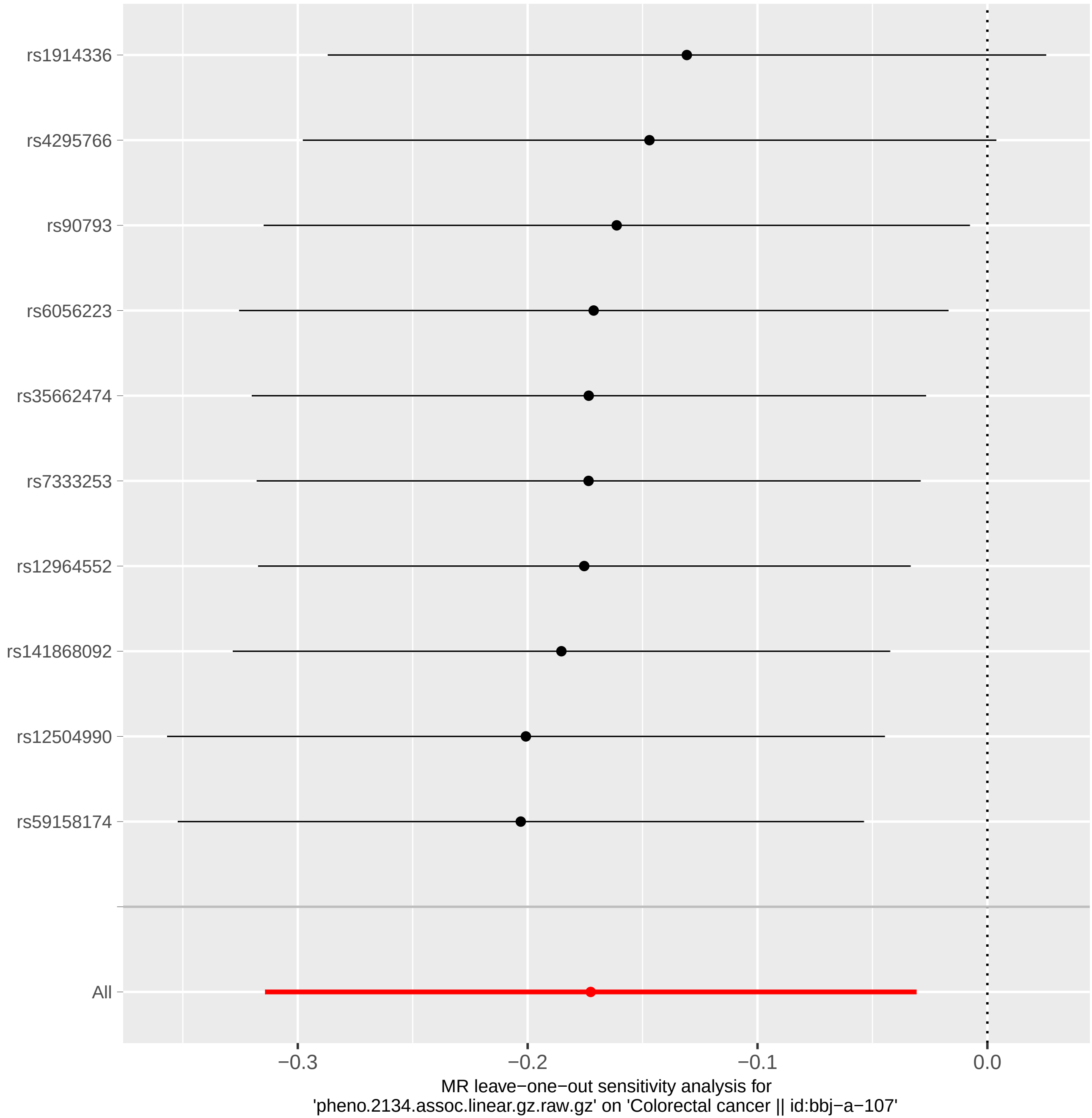
rs1914336
rs4295766
rs90793
rs6056223
rs35662474
rs7333253
rs12964552
rs141868092
rs12504990
rs59158174
All
−0.3
−0.2
−0.1
0.0
MR leave−one−out sensitivity analysis for
'pheno.2134.assoc.linear.gz.raw.gz' on 'Colorectal cancer || id:bbj−a−107'
